# How Do Children Evaluate Scientific Explanations Provided by Digital Voice Assistants, Teachers, and Peers?

**DOI:** 10.3390/bs16050661

**Published:** 2026-04-27

**Authors:** Amanda S. Haber, Sona C. Kumar, Melia Swenson, Kara Bode, Elizabeth Ruel

**Affiliations:** 1 Department of Psychological and Brain Sciences, Meditz College of Arts and Sciences, Fairfield University, 1073 North Benson Road, Fairfield, CT 06824, USA; 2 Department of Psychology, Davidson College, 425 Concord Road, Davidson, NC 28036, USA

**Keywords:** digital tools, early childhood education, STEM learning

## Abstract

As of 2025, there are approximately 154.3 million voice assistant users in the United States (Emarketer, 2025). Given the prevalence of digital voice assistants in children’s lives, it is critical to understand how children interact with and learn from such digital technologies. Across two experiments, we utilized a modified selective trust design to explore children’s (*N* = 310) information-seeking behaviors towards technological and human sources in the science domain. In Experiment 1 (*N* = 143), we asked whether children (aged 4–6) are more likely to direct scientific questions towards and trust in scientific explanations from a digital voice assistant or a peer. The experiment included three parts: (i) *scientific ask and endorse phase* (ii) *explicit judgement phase* and (iii) *digital voice assistant familiarity question phase*. In the first part of the *scientific ask and endorse* phase, children were asked who they would rather ask to answer certain scientific questions. In the second part of this phase, the digital voice assistant and the peer each provided an explanation in response to that question. Half of the children were assigned to a condition where the digital voice assistant provided a noncircular explanation, and the other half of the children were assigned to a condition where the peer provided a noncircular explanation. In Experiment 2 (*N* = 167), we examined children’s preference to pose scientific questions to and trust in explanations from a digital voice assistant or a classroom teacher. Across both studies, children preferred to ask questions and trust scientific explanations from the digital voice assistant rather than the peer or the teacher. By understanding how children learn with and through digital technologies in the domain of science, we can design future interventions that leverage conversational AI to further enhance children’s science engagement and critical thinking skills during the early childhood years.

## 1. Introduction

iPad, iPhone, iThis and iThat. Although children can acquire information through their direct experimentation with the world around them, children growing up in the digital age learn a great deal of information about the world by directing scientific questions (e.g., “why do fish live underwater?”) to people (e.g., teachers, caregivers, and peers) as well as technological sources of information including the internet, robots, and digital voice assistants/smart speakers (e.g., Apple’s *Siri*, Amazon’s *Alexa*, Google Home; e.g., Aeschlimann et al., 2020; Girouard-Hallam & Danovitch, 2022; Girouard & Danovitch, 2026; Hoehl et al., 2024; Lovato et al., 2019; Murray, 2021; Stower et al., 2021; Tong et al., 2025; Vella et al., 2025; Wang et al., 2019; Wu et al., 2024). Arguably, children’s scientific “conversations” (question–response exchanges) with human informants or digital technologies can shape their STEM learning during the early childhood years (e.g., Bonus et al., 2025; Callanan et al., 2020; Haber et al., 2021a, 2021b; Haber, 2025; Haden et al., 2023; Kelly et al., 2023). Currently, children (ages 0–8) spend approximately 2 h and 27 min per day interacting with screen media (Common Sense Media, 2025). Their engagement only increases with age, with 5–8-year-olds spending on average over three hours and twenty-eight minutes interacting with screen media per day (Common Sense Media, 2025). According to the Common Sense Media (2025) report, 96% of American children live in a home with at least one smartphone device and 41% of American children have a digital voice assistant (smart speaker) at home, an 8% increase from 2020. Indeed, “by 2 years old, 4 in 10 children have their own tablet (40%) and by 4 years old, more than half of children do (58%)” (Common Sense Media, 2025, p. 4). Moreover, over 21% of caregivers reported that children use smart speakers at least a few times a week, most often to obtain information and play music (Common Sense Media, 2025). Taken together, these data suggest that children are both exposed to and actively use technological sources from an early age.

Yet, we know very little about children’s preference for digital voice assistants as scientific informants and the extent to which they trust their explanations. Children’s early STEM learning, especially their learning about unobservable scientific processes such as germs or electricity, often relies on scientific explanations from more knowledgeable others. Today, a more knowledgeable other may include not only a caregiver, teacher, and peer, but also technology (Canfield & Ganea, 2014; Clegg et al., 2019; Haber et al., 2022; Harris et al., 2018; Leech et al., 2020; Vygotsky, 1978; Willard et al., 2019). In the current set of experiments, we ask how young children decide to direct scientific questions to digital voice assistants and how they trust scientific explanations provided by such sources.

Digital voice assistants or smart speakers represent a unique source of information. According to Common Sense Media (2020), such technologies are defined as “any device that can respond to verbal commands, play music, and answer questions” (p. 12). Whereas interaction with computers or the internet may require the ability to read or write, children can interact with digital voice assistants directly in similar ways to with human informants or robots (Lau et al., 2018; Szczuka et al., 2022; Wojcik et al., 2022). Indeed, children are likely to associate digital voice assistants with robots (Common Sense Media, 2025; Wojcik et al., 2022). Despite the paucity of empirical data exploring children’s evaluation of information from digital voice assistants, previous research has explored children’s interactions with social robots (Breazeal et al., 2016; Cameron et al., 2016, 2017; Di Dio et al., 2020a, 2020b; Goldman et al., 2023; Hoehl et al., 2024; Manzi et al., 2020; Melson et al., 2009; Sommer et al., 2019, 2021a, 2021b, 2023; Tanaka et al., 2007; Van Straten et al., 2023) and, more recently, Artificial Intelligence (Oh et al., 2025). Findings indicate that children seem to consider cues such as accuracy, agency (e.g., intentionality) and experience (Brink & Wellman, 2020; Cameron et al., 2016, 2017; Hoehl et al., 2024; Manzi et al., 2020; Melson et al., 2009). For example, 3-year-olds prefer to learn from an accurate social robot over an inaccurate robot, and 5-year-olds prefer information provided by a reliable humanoid robot (Brink & Wellman, 2020; Hoehl et al., 2024). Overall, research suggests that as children get older, they are less likely to view robots as social beings (attributing feelings or intentions to machines, e.g., Cameron et al., 2016, 2017; Hoehl et al., 2024; Manzi et al., 2020).

When a child interacts with a digital voice assistant, their primary way of acquiring knowledge involves asking questions (Danovitch et al., 2025). Given that preschool children ask over 76 information-seeking questions per hour (Chouinard, 2007; Kurkul & Corriveau, 2018; Ronfard et al., 2018) and continue to persist when they are not satisfied with the response (Frazier et al., 2016), it is critical to understand how children view such digital voice assistants as sources of information. In one study, Lovato et al. (2019) provided 18 families (with children aged 5–6) with a smart speaker (*Alexa*) and found that children pose questions to smart speakers about a variety of topics including science and technology (24% of questions), practical items (weather and directions; 18%) and personal information (9%). Similarly, the findings from Beneteau et al. (2019), Druga et al. (2017), and Yarosh et al. (2018) provide further evidence that young children are directing questions to such devices, and children seem to revise questions in order obtain the accurate answer from digital voice assistants (Danovitch et al., 2025; Festerling & Siraj, 2020; Girouard-Hallam & Danovitch, 2022). In other work, Wojcik et al. (2022) examined 5- and 6-year-olds’ decision to learn information about obscure animals from a digital voice assistant (*Amazon’s Echo*) or a human informant. Although the study included a small sample size (approximately 30 children), the findings demonstrate that the children were more likely to direct the questions to a digital voice assistant rather than the human informant. However, they demonstrated no significant preference in endorsing information from either informant. Finally, research indicates that children use digital voice assistants to inquire about factual rather than personal information (e.g., Lovato et al., 2019; Oranç & Ruggeri, 2021). Recently, Girouard-Hallam and Danovitch (2022) found that older children (7–8-year-olds) were more likely than younger children (4–5-year-olds) to seek factual information from a digital voice assistant rather than a human informant. Taken together, these data suggest that children are increasingly engaging with digital technologies from a young age, and digital voice assistants can shape their learning during early childhood.

- The Current Study

As of 2025, there are approximately 154.3 million voice assistant users in the United States, and this number is anticipated to grow to at least 170.3 million users by 2028 (Wohr, 2025). Given the prevalence of digital voice assistants in children’s lives, it is critical to understand children’s preference for and trust in explanations from such technological sources of information. This is particularly important in the domain of science, as the quality of scientific explanations can impact children’s early STEM learning (e.g., Danovitch et al., 2021; Leech et al., 2020; Mills et al., 2022; Russ et al., 2008; Sands et al., 2023; Venkadasalam et al., 2024). Yet, to the best of our knowledge, little research has examined whom children decide to direct scientific questions to and their trust in scientific information provided by such sources.

Across two studies, we utilized a modified selective trust design (see Harris et al., 2018 for review) to explore children’s preference for directing scientific questions to and epistemic trust in explanations provided by technological and human sources in the science domain. For the purposes of this work, *epistemic trust* is defined as children’s expectations that a source will provide an accurate explanation. Findings from an extensive body of research indicate that children often endorse information from sources who have been accurate or demonstrate expertise (e.g., Koenig & Harris, 2005; Pasquini et al., 2007; Sobel & Corriveau, 2010).

In Experiment 1, we examined children’s preference for directing scientific questions to and their trust in scientific explanations provided by a digital voice assistant or a peer (e.g., Wang et al., 2019). In line with previous research, the peer was described as “a child just like you.” We focus on the digital voice assistant versus a peer in Experiment 1 because 4-year-olds have not yet started kindergarten, and therefore, they may have less experience with classroom teachers compared to 5- and 6-year-olds.

The experiment included three parts: (i) *scientific ask and endorse phase* (ii) *explicit judgement phase* and (iii) *digital voice assistant familiarity question phase*. Adapted from Wang et al. (2019), we first measured children’s preference for directing scientific questions from either source before providing any explanation (*scientific ask trials*). Children’s epistemic trust was operationalized based on which informant’s explanations they endorsed (*scientific endorse trials*).

In the first part of the *scientific ask and endorse phase*, children are asked who they would rather ask to answer certain scientific questions (for example, “If you are going to find out why it rains, which would you ask, Alexa/Siri/Google or the child? Why”).

In the second part of this phase, the digital voice assistant and the peer each provided an explanation in response to that question. In the science domain, scientific explanations can shape children’s understanding about concepts and mechanisms that underlie causal processes (Callanan & Oakes, 1992; Legare & Lombrozo, 2014; Leech et al., 2020; Willard et al., 2019). Research indicates that with age, children become more sensitive to the quality of the explanations that they receive from others (Frazier et al., 2009; Kurkul & Corriveau, 2018; Wu et al., 2024). One factor that contributes to explanation quality is circularity, which can have a significant impact on science learning (Corriveau & Kurkul, 2014; Clegg et al., 2019, Ma et al., 2024; Mercier et al., 2014; Mills et al., 2019; Mills et al., 2022; Sands et al., 2023; Wu et al., 2024). Noncircular explanations typically provide a logical explanation to a question (e.g., “when the light is turned on the circuit is complete and that allows electricity to flow to the light”; Corriveau & Kurkul, 2014). Circular explanations, which are common in everyday conversation, typically reiterate the information in the question around in a circle (e.g., “the lights turn on because the switch is turned on, and it gives us light”; Corriveau & Kurkul, 2014). Research indicates that as young as four years of age, children prefer noncircular rather than circular explanations (Corriveau & Kurkul, 2014; Mills et al., 2019) and such information can impact children’s decision to learn new information from the source (e.g., Baum et al., 2008; Corriveau & Kurkul, 2014; Mercier et al., 2014; Wu et al., 2024). In our study, half of the children were assigned to a condition where the digital voice assistant provided noncircular explanations, and the other half of the children were assigned to a condition where the peer provided noncircular explanations.

In the *explicit judgement phase*, children are asked to determine who is “better” at answering science questions. Finally, in the *familiarity question phase*, children are asked about their use of digital voice assistants at home.

We predicted that children may prefer to direct scientific questions to the digital voice assistant (regardless of explanation quality). For example, Wang et al. (2019) found that children demonstrated a preference for seeking scientific and historical information provided by the internet (Wang et al., 2019), suggesting that as children get older, they may prefer more technological sources of information. Similarly, Girouard and Danovitch (2026) found that with age, 4- to 8-year-olds endorse information provided by Google at higher rates compared to information provided by a teacher. We had no specific predictions about how children would evaluate the quality of the explanations provided by the digital voice assistant versus the peer.

In Experiment 2, we utilized the same methodology as Experiment 1 to examine children’s preference for a digital voice assistant or a classroom teacher.

## 2. Experiment 1

### 2.1. Materials and Method

#### 2.1.1. Participants

The first study involved 143 4-, 5- and 6-year-old children (*M_age_* = 64.72 months, *SD* = 10.13 months, range = 48–83 months, 76 girls, 67 boys) and 143 caregivers (138 mothers, 4 fathers, 1 nonbinary). In line with similar research (Girouard-Hallam et al., 2021; Girouard-Hallam & Danovitch, 2022) and a priori analyses conducted using G*power 3.1.9.7 (Faul et al., 2007), we aimed to recruit a sample of at least 129 children to conduct logistic regression models with binary dependent variables (the analysis with the greatest number of parameters; power = 0.95; small effect size ƒ = 0.15) (see Supplementary Materials). Thirty additional children participated in the experiment but were excluded from analyses due to failure on the memory check explicit judgement question (*n* = 14) or significant missing data (*n* = 16). As illustrated in Table 1, approximately 42.7% of children were non-White (note: *n* = 2 caregivers did not report the child’s race and ethnicity). Further, Table 2 includes demographic information for caregivers (e.g., highest education level and income level). Informed consent was obtained according to the Institutional Review Board.

#### 2.1.2. Procedure

Families were recruited to participate through a laboratory database, public advertisements on social media, and flyers at libraries and childcare centers in Northeastern cities in the United States. The study was approved from the Institutional Review Board at Fairfield University and informed consent was obtained. All families received a $10 gift card for their participation.

Children first joined a Zoom session with an experimenter using a computer or tablet. Although caregivers remained in the room, they were instructed not to engage in any communication with their child during the session (Girouard-Hallam et al., 2021). The study included the following phases presented in a fixed order:

**Digital Voice Assistant Introduction**: To start, the experimenter provided the child with a brief description of digital voice assistants (design adapted from Girouard-Hallam et al., 2021): “These devices are speakers that can respond to the sound of your voice and can answer your questions. The devices can all come in different shapes, colors, and sizes.” Next, the experimenter showed the child photos of three voice assistants: “Alexa” (Amazon), “Siri” (Apple)” and “Google Home” and asked the child which one they “knew the most about.” The order of the three voice assistants was counterbalanced across participants. The following questions focused on the digital voice assistant (DVA) the child was most familiar with.

**Science Ask and Endorse Phase**: The experimenter began by explaining to the child that “Today you are going to hear some science questions. A ‘child like you’ (adapted from Wang et al., 2019; experimenter pointed to a picture of a peer) or [Alexa/Siri/Google] (a photograph of the digital voice assistant the child was most familiar with was used for the study) can help us find the answer to these questions. Let’s see what science questions we have for today!” There were five science topics: airplanes, electricity, fish, rain, and flowers/trees (see Table 3).

For each science topic, there was one *Ask* question (always presented first) and one *Endorse* question (always presented second). There were five total “Ask Questions” and five total “Endorse Questions.” For the *Ask* question, the experimenter asked the child to identify who they would rather ask to answer a science question. For example, the experimenter said, “*If you are going to find out how fish breathe underwater, which would you ask, [Alexa/Siri/Google] or the child?*” Children were asked to justify their response.

For the *Endorse* question, the DVA and the peer each provided an explanation in response to the specific science question (read by the experimenter). Half of the children were assigned to a condition where the DVA always provided a noncircular explanation (*n* = 71), and the other half were assigned to a condition where the peer always provided a noncircular explanation (*n* = 72; see Table 3 for examples of explanations). Whereas circular explanations reiterate the information in the question around in a circle (e.g., “the lights turn on because the switch is turned on, and it gives us light”), noncircular explanations provide a logical explanation to a question (“lights turn on because the circuit is complete, and electricity flows to turn on the light”; Corriveau & Kurkul, 2014; Wu et al., 2024). For example, the experimenter said, “[Alexa/Siri/Google] says fish take in water through their gills and dissolve oxygen from water into their blood. [The child] says fish breathe under the water and fish do not live or move around on land.” After hearing the explanations, the experimenter asked, “Do you like what [Alexa/Siri/Google] or the child said?” Again, children were asked to justify their response. The order of the science topics and the explanations were counterbalanced across participants (Corriveau & Kurkul, 2014).

**Explicit Judgement**: In the “Explicit Judgement” phase, children were presented with a memory check question (“do you remember when Alexa/Siri/Google and the child were talking about some things that we know like why it rains and how airplanes fly?”). If children did not respond to the question or answered “no,” they were removed from the dataset (Corriveau & Kurkul, 2014). In line with past selective trust research, children were asked three explicit judgement questions. The experimenter asked the child, “was [Siri/Alexa/Google] very good or not very good at explaining those things?” and “was the child very good or not very good at explaining those things?” The order of these questions was counterbalanced across participants. Finally, children were asked, “who do you think is better at answering science questions?” For each of the three explicit judgement questions, children were asked to justify their responses.

**Familiarity Questions**: Finally, adapted from past research (Girouard-Hallam et al., 2023) during the “Familiarity Questions” phase, the child was asked about their experience using DVAs (e.g., “Do you use it to play games or listen to music?”). Additionally, the caregiver also completed a familiarity questionnaire after the Zoom session to gain an understanding of DVA use in their house (parent survey items adapted from Girouard-Hallam & Danovitch, 2022). The familiarity questionnaire was collected as part of a larger project on children’s use of digital voice assistants, which is outside the scope of the current experiments.

#### 2.1.3. Coding

**Justification Coding for Science Ask and Endorse Trials:** Children were invited to justify their response for the five science *Ask* questions and five science *Endorse* questions. Justification responses were transcribed and coded into ten mutually exclusive categories based on themes that emerged in the data (see Table 4 for full coding scheme and examples): *Attribute*, *Knowledge*, *Prior Knowledge*, *Making Sense/Understand*, *Truth/Right*, *Repetition*, *Better*, *Status as Informant*, *I don’t know*, and *No answer/Nonsense*.

**Justification Coding for Explicit Judgement Questions:** Children were asked to justify their response for three *explicit judgement* questions, which were coded into ten mutually exclusive categories based on themes that emerged in the data (see Table 5 for full coding scheme and examples): *Attribute*, *Knowledge*, *Prior Experience*, *Explanation Quality*, *Accuracy/Inaccuracy*, *Repetition*, *Better*, *Status as Informant*, *I don’t know*, and *No Answer*.

To confirm inter-rater reliability, a random sample of 127 responses (16.5% of the total sample) was coded by two trained research assistants. Agreement was at 99% (Kappa = 0.99). Disagreements were resolved through discussion with the first author.

### 2.2. Results

#### 2.2.1. Preliminary Results

Overall, 48.3% of children were most familiar with *Alexa*, 18.2% with *Google Home*, and 33.6% with *Siri*. Importantly, a preliminary chi-square test of independence indicated that there was no difference in children’s familiarity with DVAs by condition (*X*^2^(1) ≈ 0, *p* = 1).

#### 2.2.2. Initial Science Ask Preference

We first conducted a binomial test to determine whether the proportion of children’s initial preference (before either informant provided an explanation) for whether to ask the DVA or the peer a science question differed from chance (0.5). Across conditions, children significantly preferred the DVA over the peer when asked at the beginning of the study (*p* < 0.001). Additionally, a logistic regression controlling for condition did not show any age-related differences in children’s initial ask preference (see Table 6).

#### 2.2.3. Initial Science Endorsement

We conducted a binomial test to determine whether the proportion of children’s initial endorsement of the DVA or the peer’s science explanation differed from chance (0.5). Across conditions, children’s initial informant endorsement differed from chance, with significantly more children endorsing the DVA over a child (*p* < 0.001). Of children who endorsed the DVA, there was no difference by explanation quality (*p* = 0.68). Of children who endorsed the peer, there was also no difference by explanation quality (*p* = 0.67). There were no age-related differences in children’s initial endorsement, holding condition constant (see Table 6).

#### 2.2.4. Total Science Ask Preference

Across five trials, children were asked which informant they would ask a science question. Children were categorized as either showing a DVA preference (asking the DVA in three or more trials) or as showing a peer preference (asking the peer in three or more trials). One child showed no preference and was excluded from this analysis. An exact binomial test indicated that across conditions, children showed a DVA preference at above chance levels (*p* < 0.01). Of children who preferred the DVA, there was no difference by condition (*p* = 0.62); similarly, of children who preferred the peer, there was no difference by condition (*p* = 0.42). A logistic regression controlling for condition found no difference in children’s preference by age (see Table 6). See Table 7 for justification responses.

#### 2.2.5. Total Science Ask Endorsement

Across five trials, children were asked which informant’s scientific explanation they would endorse. Children were categorized as either showing a DVA preference (endorsing the DVA in three or more trials) or as showing a peer preference (endorsing the peer in three or more trials). One child showed no preference and was excluded from this analysis. An exact binomial test indicated that across conditions, children showed a DVA preference at above chance levels (*p* < 0.01). Of children who preferred the DVA, there was no difference by condition (*p* = 0.12). Of children who preferred the peer, more children showed this preference when the peer provided a noncircular than a circular explanation (*p* = 0.02). A logistic regression controlling for condition found no difference in children’s preference by age (see Table 6). See Table 7 for justification responses.

#### 2.2.6. Explicit Judgement

Children were invited to judge the peer and DVA explanations as either good or not very good. We then examined whether children’s judgements differed by explanation quality.

**DVA:** A binomial exact test showed that across conditions, the DVA explanations were more likely to be judged as good than not good by children (*p* < 0.01). This held true regardless of explanation quality. When DVA explanations were circular (*p* < 0.001) and noncircular (*p* < 0.001), children were more likely to judge them as good.

**Peer:** A binomial exact test showed that across conditions, there was no difference from chance in children’s rating of the peer as good or not very good (*p* = 0.93). Further analysis revealed that when the peer provided a circular explanation, children’s rating of the peer as good or not very good was at chance (*p* = 0.48). When the peer provided a noncircular explanation, children also were at chance in judging the peer’s explanations as good or not very good (*p* = 0.29).

**DVA versus Peer:** Finally, children were asked to judge who was better, the DVA or the peer. Across conditions, the DVA was rated as better than the peer (*p* < 0.001). For those who rated the DVA better, there was no difference by explanation quality (*p* = 0.69); for those rated the peer better, there was also no difference by explanation quality (*p* = 0.75). See Table 7 for justification responses.

### 2.3. Experiment 1 Discussion

In Experiment 1, we examined children’s preference for directing scientific questions to a digital voice assistant versus a peer. We also explored children’s trust in explanations provided by such sources of information. We briefly discuss the findings below.

First, 4-, 5-, and 6-year-olds demonstrated a preference for directing scientific questions to and trust in scientific explanations provided by a digital voice assistant rather than a peer. Overall, the findings from *the science ask and endorse trials* provide support for our hypothesis, adding to evidence from past research (Beneteau et al., 2019; Druga et al., 2017; Girouard-Hallam & Danovitch, 2022; Lovato et al., 2019; Oranç & Ruggeri, 2021; Wojcik et al., 2022) suggesting that children direct questions to digital voice assistants in the science domain (viewing such devices as a critical sources of information). Our findings also indicate that 4-, 5- and 6-year-old children not only *prefer* DVAs as scientific informants (*science ask trials*), but they also seem to *trust* their explanations (*science endorse trials*). Further, when asked to justify who was “better” at answering science questions (*global evaluation*), across conditions, children rated the DVA as better than the peer.

One possible explanation might be that children viewed the digital voice assistants as having more expertise and knowledge compared to their peers (e.g., Wang et al., 2019). Indeed, we found that regardless of explanation quality (noncircular versus circular explanations), children trusted explanations provided by digital voice assistants. Therefore, in order to better understand how children evaluate scientific information in the world around them in the digital age, we also wanted to examine children’s trust when faced with scientific information from a classroom teacher versus a digital voice assistant. Arguably, a classroom teacher would have more expertise about concepts and processes in the science domain than a child. Therefore, it is plausible that when faced with informants with similar expertise, children may consider the quality of the explanation when deciding whom to trust for scientific information.

## 3. Experiment 2

### 3.1. Introduction

Given the findings from Experiment 1, for Experiment 2, we examined children’s preference to pose scientific questions to and trust scientific explanations provided by the digital voice assistant or a classroom teacher (instead of the peer).

We had several hypotheses for Experiment 2. First, we anticipated that given the findings from Experiment 1 and past work, children would prefer to direct scientific information to the digital source rather than the classroom teacher (e.g., Girouard & Danovitch, 2026; Wang et al., 2019). We had two plausible explanations for children’s trust in scientific explanations provided by digital voice assistants versus a classroom teacher. On the one hand, given the findings from Experiment 1, it seemed possible that children would trust scientific explanations provided by digital voice assistants (regardless of explanation quality) rather than the classroom teacher. On the other hand, it also seemed plausible that children would be more likely to endorse the classroom teacher when the teacher provided a noncircular rather than a circular explanation. As highlighted above, findings from past research indicate that children (aged 4–10) prefer the informant who provides noncircular (versus circular) explanations (Corriveau & Kurkul, 2014; Mills et al., 2019; Wu et al., 2024).

### 3.2. Materials and Method

#### 3.2.1. Participants

The second study involved 167 4-, 5- and 6-year-old children (*M_age_* = 65.44 months, SD = 10.66 months, range = 48–83 months, 97 girls, 69 boys, 1 nonbinary) and 167 caregivers (159 mothers, 8 fathers). Twenty-two additional children participated in the study but were excluded from analyses due to failure on the memory check question (*n* = 20) or no familiarity with digital voice assistants (*n* = 2). As illustrated in Table 8, approximately 44.3% of children were non-White (note: *n* = 1 caregiver did not report the child’s race and ethnicity). Table 9 includes demographic information for caregivers (e.g., highest education level and income level). Informed consent was obtained according to the Institutional Review Board.

#### 3.2.2. Procedure and Coding

The procedure and the coding for open-ended justifications were identical to Experiment 1, with the exception of one informant. Instead of choosing between a DVA and a peer, children were invited to choose between a DVA and a classroom teacher. The experimenter began by explaining to the child that “Today you are going to hear some science questions. A teacher (experimenter pointed to a picture of a teacher) who teaches in your school or [Alexa/Siri/Google] (a photograph of the digital voice assistant the child was most familiar with was used for the study) can help us find the answer to these questions. Let’s see what science questions we have for today!”

Half of the children were assigned to a condition where the DVA always provided noncircular explanations (*n* = 80), and the other half were assigned to a condition where the classroom teacher always provided noncircular explanations (*n* = 87). All explanations were identical to Experiment 1 (see Table 3).

### 3.3. Results

#### 3.3.1. Preliminary Results

Overall, 41.9% of children were most familiar with *Alexa*, 23.4% with *Google Home*, and 34.7% with *Siri*. Importantly, a preliminary chi-square test of independence indicated that there was no difference in children’s familiarity with DVAs by condition (*X*^2^(1) = 0.49, *p* = 0.5).

#### 3.3.2. Initial Science Ask Preference

We conducted a binomial test to determine whether the proportion of children’s initial preference (before either informant provided an explanation) for whether to ask the DVA or the teacher a science question differed from chance (0.5). The results indicated that children’s initial preference differed from chance, with significantly more children preferring to ask the DVA rather than the teacher across conditions (*p* < 0.01). Finally, we conducted a logistic regression to determine whether there were differences in children’s informant preference by age in years, holding condition constant. Age did not predict informant preference (see Table 10).

#### 3.3.3. Initial Science Endorsement

We conducted a binomial test to determine whether the proportion of children’s initial endorsement of the DVA or teacher’s science explanation differed from chance (0.5). The results indicated that children’s initial preference differed from chance, with significantly more children endorsing the DVA than the teacher (*p* < 0.001). We then split the data into children who chose the DVA and children who chose the teacher in order to examine whether there were differences in children’s decision-making depending on whether the informant’s explanation was circular or noncircular. Of children who chose the DVA, there was no difference in their preference based on explanation quality (*p* = 0.10). Of children who chose the teacher, there was a significant difference from chance (0.5) in preference based on explanation quality (*p* < 0.01). In other words, children were more likely to endorse the teacher when the teacher provided a noncircular explanation than a circular explanation. Finally, we conducted a logistic regression to determine whether there were differences in children’s informant preference by age in years, holding condition constant. Age did not predict informant preference (see Table 10).

#### 3.3.4. Total Science Ask Preference

Across five trials, children were asked which informant they would rather ask a science question. Children who chose to ask the DVA in three or more trials were coded as showing a DVA preference; children who endorsed the teacher in three or more trials were coded as showing a teacher preference. One child showed no preference and was excluded from this analysis. We conducted a binomial test to determine whether the proportion of children with a DVA preference compared to a teacher preference differed from chance (0.5). The results indicated that children’s preference differed from chance, with significantly more children endorsing the DVA than the teacher (*p* < 0.001). We then split the data into children who chose the DVA and children who chose the teacher in order to examine whether there were differences in children’s decision-making depending on whether the informant provided a circular or noncircular explanation. Of children who chose the DVA, there was no difference in their preference based on explanation quality (*p* = 0.18). Of children who chose the teacher, there was a significant difference from chance (0.5) in preference based on explanation quality (*p* < 0.01). Specifically, children were more likely to prefer the teacher when the teacher provided a noncircular explanation than a circular explanation. A binary logistic regression controlling for condition indicated that age did predict preference, with six-year-olds showing a stronger preference for the teacher than four-year-olds (see Table 10). See Table 11 for justification responses.

#### 3.3.5. Total Science Ask Endorsement

Across five trials, children were asked which informant’s scientific explanation they would endorse. Children who endorsed the DVA in three or more trials were coded as showing a DVA preference; children who endorsed the teacher in three or more trials were coded as showing a teacher preference. We conducted a binomial exact test to determine whether the proportion of children with a DVA preference compared to a teacher preference differed from chance. The results indicated that children’s preference differed from chance significantly, with more children endorsing the DVA over the teacher (*p* < 0.001). We then examined how explanation quality impacted children’s decision-making. Of children who endorsed the DVA, children showed a preference for when the DVA provided a noncircular explanation rather than a circular explanation (*p* < 0.01). Of children who endorsed the teacher, children were more likely to endorse them when the teacher provided a noncircular explanation than a circular explanation (*p* < 0.001). A binary logistic regression controlling for condition indicated that age did predict informant preference, with six-year-olds showing a stronger preference for the teacher than four-year-olds (Table 10). See Table 11 for justification responses.

#### 3.3.6. Explicit Judgement

Children were invited to judge the teacher and DVA explanations as either good or not very good. We then examined whether children’s judgements differed by explanation quality.

**Teacher:** A binomial exact test showed that across conditions, the teacher explanations were judged as good by children (*p* < 0.01). Further analysis revealed that when the teacher provided a circular explanation, children’s rating of the teacher as good or not very good was at chance (*p* = 1). When the teacher provided a noncircular explanation, children were more likely to rate the teacher’s explanation as good (*p* < 0.001).

**DVA:** A binomial exact test showed that across conditions, the DVA explanations were judged as good by children (*p* < 0.01). When the DVA provided a circular explanation, children were more likely to rate the DVA explanations as good than not good (*p* < 0.01). When the DVA provided a noncircular explanation, children were also more likely to rate the DVA explanations as good than not good (*p* < 0.001).

**DVA versus Teacher:** Finally, children were asked to judge who was better, the DVA or the teacher. Across conditions, the DVA was rated as better than the teacher (*p* < 0.01). In Condition 1, when the DVA provided a noncircular explanation, children rated the DVA as better than the teacher (*p* < 0.001). In Condition 2, when the teacher provided a noncircular explanation, children’s responses were at chance (*p* = 1). See Table 11 for justification responses.

### 3.4. Experiment 2 Discussion

In Experiment 2, we examined children’s preference for directing scientific questions to a digital voice assistant versus a classroom teacher. We also examined if children evaluate the quality of the explanation when deciding to trust scientific information provided by such sources of information. Overall, the findings from Experiment 2 provide further support for the results in Experiment 1.

First, across the *science ask and endorse questions*, 4-, 5- and 6-year-olds, preferred to direct scientific questions to the digital voice assistant rather than the classroom teacher, suggesting that children view digital voice assistants as sources of information in the science domain (Beneteau et al., 2019; Druga et al., 2017; Girouard-Hallam & Danovitch, 2022; Lovato et al., 2019; Oranç & Ruggeri, 2021; Wojcik et al., 2022). In the General Discussion, we turn to this point again.

Second, we hypothesized that children would be more likely to endorse the explanation provided by the classroom teacher when the teacher provided the noncircular (higher-quality scientific) rather than the circular explanation. In Experiment 2, we found some support for this hypothesis: children who endorsed the teacher were more likely to do so when the teacher provided the noncircular explanation during the *initial science endorsement, total science ask preference* and *total science endorsement trials*. Although we did not have specific predictions about DVAs and explanation quality, we also found in Experiment 2 that children who endorsed the DVA were more likely to do so when the DVA provided the noncircular rather than the circular explanation during the *total science endorsement and explicit judgement: DVA versus teacher.* This is somewhat consistent with past research findings that children (aged 4–10) preferred the informant who provided higher-quality noncircular (versus circular) explanations (Corriveau & Kurkul, 2014; Mills et al., 2019; Wu et al., 2024). Importantly, in past work (see Corriveau & Kurkul, 2014), human informants provided the noncircular and circular explanations. One explanation for our findings is that children may consider explanation quality as a cue of credibility when deciding whom to trust for scientific information provided by human informants, but not digital technologies. We return to this point again in the General Discussion.

## 4. General Discussion

In a world of digital media, children are exposed to and actively use digital technologies; in turn, this is rapidly transforming how children acquire knowledge during early childhood, especially in the science domain. Across two experiments, we find that young children display a significant preference for learning scientific information from a digital source, which as we discuss below, is one way in which digital media can shape children’s STEM learning during the early years.

First, the results from Experiments 1 and 2 indicate that 4-, 5-, and 6-year-old children prefer digital voice assistants as scientific informants (preferring to direct scientific questions to them) and trust their scientific explanations over peers (Experiment 1) or classroom teachers (Experiment 2). These data support our first hypothesis, providing further evidence that as young as 4 years of age, children seem to view digital voice assistants as a source of information in the science domain, preferring to seek and trust scientific information provided by such digital technologies (Aeschlimann et al., 2020; Beneteau et al., 2019; Druga et al., 2017; Girouard-Hallam & Danovitch, 2022; Lovato et al., 2019; Oranç & Ruggeri, 2021; Wang et al., 2019; Wojcik et al., 2022).

Second, the findings from Experiments 1 and 2 suggest that children seem to ignore explanation quality from digital voice assistants. In other words, children often trusted the scientific explanations provided by a digital voice assistant over a peer or a classroom teacher, regardless of whether the DVA provided a noncircular or circular explanation.

One plausible explanation for these findings is that children view digital voice assistants as familiar informants. In our study, the DVA was selected based on the device children “know the most about”, ensuring baseline familiarity. In contrast, the teacher (described as a “teacher who teaches in your school”) and the peer (described as a “child like you”) were both represented by pictures, rather than real individuals. As a result, the familiarity manipulation was not symmetric across informants, which may have influenced children’s preferences. Although there were no significant differences in children’s familiarity with DVAs by condition for either experiment, we found that children are most familiar with *Alexa*, followed by *Siri* and finally, *Google Home*. Perhaps, children’s willingness to direct questions to DVAs is influenced by how the informants were presented, and their familiarity with such devices (e.g., Lovato & Piper, 2015; Druga et al., 2017).

However, arguably, children’s familiarity with the DVAs may increase their use of them but may not influence how they evaluate the accuracy of information provided by such devices. First, children’s familiarity with digital voice assistants may focus more the “function” of such devices. From an early age, children may learn how to engage with DVAs, which often involves providing fast responses to questions. This may suggest that children’s familiarity with such devices is based on understanding of their function, not why the devices might know information. Whereas children’s familiarity with human informants may reflect an understanding that people have desires, intentions, expertise and minds, their familiarity with DVAs may enhance their use of such devices, but not their expectations about expertise or explanation quality. Second, previous research indicates that children are more likely to seek factual or procedural information rather than personal or social information from DVAs (e.g., Girouard-Hallam & Danovitch, 2022). If familiarity was the only reason children prefer such devices, their preference and trust in digital voice assistants would be consistent across all domains. Our study only focused on factual and procedural questions in the domain of science, but it is plausible that for science questions related to social and personal information, children may not demonstrate this same DVA preference (Haber & Kumar, 2025). On this basis (and findings from past work), it seems that young children demonstrate a preference in turning to DVAs for information in select domains. Thus, familiarity alone cannot be the only explanation for children’s preference for digital voice assistants as scientific informants and their trust in their explanations.

A second plausible explanation is that children prefer digital voice assistants because of usability due to technology. In this case, children may prefer to turn to such devices because of the interactive and responsive nature. For example, research indicates that children may describe digital voice assistants based on function (e.g., playing music, answering questions, or setting a timer) rather than reasoning abilities or accuracy of information (e.g., Druga et al., 2017; Festerling & Siraj, 2020; Lovato & Piper, 2015; Yarosh et al., 2018). Perhaps children treat DVAs as tools, focusing on speed, and convenience of information rather than attending to the quality of the explanation or information provided by the device. This explanation could explain why children preferred digital voice assistants and trusted their explanations regardless of explanation quality.

Finally, a third plausible explanation is that children may utilize different cues of credibility when trusting information provided by digital voice assistants rather than a human informant or other inanimate objects/artifacts. Because of the interactive nature of DVAs, they may view them as demonstrating more agency than inanimate artifacts, but less cognitive capacities than humans. Recent research indicates that young children anthropomorphize DVAs, attributing social, moral and functional properties to such devices (e.g., Druga et al., 2017; Girouard-Hallam et al., 2021). For example, some young children describe DVAs as “friendly”, “helpful” or “thoughtful” (Druga et al., 2017; Girouard-Hallam et al., 2021; Yarosh et al., 2018). This type of categorization (viewing digital assistants in ways that are similar and different than human informants) may impact how they trust explanations provided by them. The findings from our study may suggest that children view digital voice assistants more as tools that provide information, but they may not always apply the same critical evaluation as they do to explanations provided by humans. Our findings from Experiment 2 suggest that when children trusted scientific explanations provided by the teacher, they were more likely to do so when teacher provided the noncircular explanation during the *initial science endorsement, total science ask preference* and *total science endorsement trials*. This may indicate that for human informants, children do use explanation quality as a cue of epistemic trust, yet they may not always apply these same criteria to digital voice assistants.

This finding has implications for explanation theories in the digital age. Arguably, the current explanation theories in cognitive and developmental psychology, which are grounded in human-to-human interactions, assume that explanations reflect beliefs and intentions and that explanations can guide learning (e.g., Keil, 2006; Lombrozo, 2006). For example, Keil (2006) argues that explanations reflect a person’s causal beliefs. In other work, Lombrozo (2006, 2016) suggests that people evaluate explanations based on evidence of what the speaker knows and their credibility (knowledge and expertise). To date, previous developmental research indicates that children use explanation quality to determine if a human informant is knowledgeable or trustworthy (e.g., Corriveau & Kurkul, 2014; Clegg et al., 2019). Whereas a noncircular explanation from a human informant may indicate more knowledge about a topic, a circular explanation from a human informant may suggest that the person is not as knowledgeable about a topic. Arguably, this process of evaluating explanation quality is focused on the beliefs and intentions of the speaker. For young children, explanations provided by digital voice assistants may not align with the argument that explanations are grounded in beliefs and intentions. It is plausible that children do not view explanations from technological devices as evidence of knowledge. On this basis, a circular explanation from a digital voice assistant may not indicate less knowledge because they may not view information from devices as reflections of cognition. Explanations from such devices may focus more on communicating information, rather than beliefs about the scientific process or concept. In other words, the scientific question–explanation exchanges between children and adults are not analogous to those of children and digital voice assistants. Although both reflect some type of “seeking and receiving information,” current explanation theories must expand to incorporate how children evaluate explanations when the “explainer” is conceptualized as an “interactive tool” (one that shares properties of human informants and inanimate objects/artifacts) rather than a human informant. This finding applies not just to digital voice assistants but to other AI-based systems as well.

Given children’s preference for digital voice assistants as scientific informants and their trust in explanations provided by such devices (regardless of explanation quality), an important step for future work is to focus on developing scaffolded interventions to foster children’s critical thinking skills (primarily the ability to apply explanation quality criteria) when engaging with digital technologies. This work is particularly important in the science domain when scientific questions and explanations can greatly transform early STEM learning (Danovitch et al., 2021; Haber et al., 2022; Leech et al., 2020; Mills et al., 2022). If children trust scientific information from digital voice assistants, it is critical that the information provided by such devices is accurate, nuanced, balanced, and correct. Given that many young children use DVAs, early childhood is a key time for children to begin learning the skills necessary to decipher misinformation and question the accuracy of scientific information provided by digital media. Further, there is more to science learning than just content: children’s understanding of science as a process (asking questions, experimenting, learning from mistakes, and sharing ideas with others), sense of belonging, interest and motivation (the relation between effort, failure and success) are all factors that contribute to children’s engagement in science (see Haber & Kumar, 2025, for review). Whereas children’s interactions with digital technologies can provide them with scientific information, children’s interactions with adults offer a unique contribution to their science engagement. Specifically, teachers and caregivers can consider the social–emotional component of science learning and provide children with hands-on scientific learning experiences. For example, through conversations about science, adults can activate children’s prior knowledge about a scientific topic or concept and encourage children to make personal connections between what they are learning about and their personal lives (e.g., Callanan et al., 2020; Haber, 2025; Haber & Kumar, 2025; Kumar & Haber, 2025; Vygotsky, 1978; Willard et al., 2019). Thus, adults continue to play a key role in fostering children’s engagement, motivation, interest, and sense of belonging in STEM. Together, digital media and human informants play complementary roles in shaping children’s early STEM experiences.

### 4.1. Digital Literacy and STEM Education: Implications for the Classroom and AI Usage in Early Childhood Education

The findings from this work also connect to research in the areas of digital literacy and STEM education. Digital literacy is defined as the skills that allow individuals to critically, locate, evaluate, interpret, and use information by digital sources (e.g., Buckingham, 2015; Lan et al., 2026; Law et al., 2018; Wei, 2022). Digital literacy is critical for children growing up in a world of technologies. According to Eshet-Alkalai’s (2004) multidimensional digital literacy framework, there are five components of digital literacy: *information literacy* (critically evaluating the quality of information), *photo-visual literacy* (understanding graphical information), *reproduction literacy* (developing new content based on exiting materials), *branching literacy* (exploring non-linear information) and *socio-emotional literacy* (comprehending social and emotional components of digital communication); see Sabyrkhanova et al. (2026). Similarly, Law et al. (2018) defined digital literacy as accessing information, critically evaluating information, creating new content and using a myriad of digital devices. Digital literacy is associated with gains in critical thinking and problem-solving skills (e.g., Tyres-Chowdhry & Binder, 2021), reading comprehension and mathematical skills and, more generally, academic achievement (e.g., Hu et al., 2018; Lan et al., 2026; see Li et al., 2025, for meta-analysis). One explanation offered by researchers for why digital literacy relates to higher academic achievement is that children and adolescents with higher digital literacy skills are able to evaluate and navigate information provided by digital technologies more efficiently and effectively. In recent years, researchers have also focused on “AI literacy,” which is considered a 21st-century digital literacy skill that focuses on effectively engaging with, communicating, and evaluating information provided by AI (e.g., Druga & Ko, 2021; Kandlhofer et al., 2016; Kewalramani et al., 2021; Long & Magerko, 2020; Ng et al., 2021; Steinbauer et al., 2021; Su et al., 2023; Williams et al., 2019; Yang, 2022). Specifically, researchers in AI literacy focus on understanding how AI systems produce outputs and critically question and evaluate information provided by such digital technologies (e.g., Long & Magerko, 2020; see Su et al., 2023 for meta-analysis).

Arguably, our study focuses on one of the fundamental AI and digital literacy skills: evaluating the quality of explanations provided by digital voice assistants. Although young children may prefer higher-quality explanations from human informants, they do not spontaneously apply the same criteria and skills to DVAs. Thus, it is critical that educators and policymakers focus on teaching skills for critically evaluating information from AI systems during the early childhood years. Such skills should include how AI systems function, limitations, and why it is critical for individuals to question the accuracy of information they receive from such systems. Indeed, according to a joint statement from the National Association for the Education of Young Children & Fred Rogers Center for Early Learning and Children’s Media at Saint Vincent College (2012), “Digital and media literacy for children means having critical viewing, listening, and web-browsing skills. Children learn to filter the messages they receive to make wiser choices and gain skills in effectively using technology and technology-and media-based information. These habits of inquiry transfer to all areas of the curriculum and to lifelong learning” (pp. 9–10). As we discuss below, STEM education provides an opportunity to teach digital and AI literacy skills to young children during the early childhood years.

Early STEM education (see National Research Council, 2013) draws on children’s natural curiosity to learn about the world around them by engaging them in core skills such as asking questions, carrying out investigations, analyzing data, constructing explanations, using evidence to support claims and communicating and evaluating information effectively. Further, according to the *Global STEM Alliance*, such 21st-century STEM skills incorporate critical thinking and problem-solving, creativity, collaboration, data literacy, digital literacy, and computer science (see Govender, 2025, for review). With the growing increase in AI systems in children’s formal and informal learning environments, researchers have started to examine the use of AI in early childhood education (e.g., Cimino et al., 2025; Ljungcrantz, 2026) and, more specifically, the use of AI in STEM education during the early years and K–12 schooling (e.g., Abisoye, 2023; Chiu & Li, 2023; Ozturk, 2025; S. J. Lee & Kwon, 2024; J. Lee, 2026). For example, Xu et al. (2022) found that integrating conversational AI into children’s narrative science programming videos was associated with higher scientific assessment scores. In other research, children’s use of programmable robots, coding platforms, and other multimedia toys is associated with computational and critical thinking skills, creativity and language development (e.g., Bourha et al., 2026; Hu et al., 2024; Kewalramani et al., 2020; J. Lee et al., 2025; Murcia et al., 2020; Papadakis, 2021; H. Q. Zeng & Ng, 2025; Zhang et al., 2025). Recently, S. A. Zeng (2025) argued that “conversational AI” (including robots, digital voice assistants, and dialogic learning apps) can be viewed as a “dialogic partner” in children’s early, play-bayed mathematical thinking and learning (focusing on four areas: cognitive scaffolding, responsiveness and timing, child agency, and math language quality). Thus, findings suggest that AI-based systems can support STEM learning in K–12 formal schooling.

Conversational AI (mainly digital voice assistants) can shape children’s STEM learning during the early childhood years. In our work, children prefer to direct scientific questions to DVAs, suggesting such AI-based digital technologies are becoming a critical social learning partner in early STEM exploration. Such interactions with DVAs can foster children’s curiosity and inquiry-based learning by encouraging them to ask questions (one of the key 21st-century STEM skills) about a scientific concept or topic (e.g., Cimino et al., 2025). In the classroom setting, DVAs can even serve as an alternative tool or aid to support STEM learning by meeting the diverse needs of students through personalized support (e.g., see Akhmetova et al., 2025, for a review of AI in high school STEM education) and by responding to questions the teacher may not be able to address due to many demands (e.g., ensuring safety (monitoring behavior) as well as curricular goals and learning objectives (Haber et al., 2021a).

Further, in the classroom context, teachers may provide a model for children of how to use DVAs as a source of information by directing questions to such devices. In K–12 education, one of the science topics includes weather and the climate (see *Next Generation Science Standards*). Let us says a teacher begins a science lesson by asking a DVA, “What is the weather today?” This step brings AI into the conversation, making it a concrete part of STEM learning (modeling question-asking behavior). However, the next step is arguably the most critical part. Rather than modeling how to ask questions and accepting the information provided by conversational AI, adults must provide a model for children of what you do once you receive this information. In our study, we saw that children trust scientific explanations provided by digital voice assistants regardless of explanation quality. Perhaps one reason we found these results is that children often observe adults direct questions to such devices, but they rarely have the opportunity to hear an adult critically evaluate the information they have just received. Thus, children may not have as much practice with watching adults engage in this next step. Rather than solely focusing on the “question-asking” aspect of such devices, we need to explicitly teach children what we do once we receive that information. Critically evaluating information is an AI and digital literacy skill, but it is also a fundamental part of the process of STEM. Let us return to the weather example. After the digital voice assistant gives an answer, the teacher may respond in a variety of ways. For example, the teacher may respond by *thinking aloud* (e.g., “I wonder where that information is coming from”), engage children by *asking a question* (“how do you think *Alexa*/Siri/*Google Home* is able to share this information with us?”) or *suggest an investigation* (“Let’s go outside and see if we can find some evidence to support that claim”). Although this is a simple example, this collaborative type of exercise provides children with a window into how AI-based systems work—inviting children to see how we can use DVAs as part of the STEM inquiry process (ask questions, investigate/analyze data, evaluate and use evidence to support explanations, and share ideas with others, all highlighted above). As the lesson and conversation continue, the teacher can explain how we might evaluate explanations from DVAs and how we determine the accuracy and credibility of information.

By bringing AI and digital literacy skills into meaningful STEM learning experiences in early childhood, we model key skills for young children including how to make observations, critically evaluate information, revise our ideas, address misconceptions, and create alternative solutions to real-world problems. These skills are particularly important in the domain of science and also create an opportunity for young children to learn about the limitations of such devices. Our findings suggest that explicit instruction is necessary for young children. Thus, in future research, we are working on designing a simple three-step intervention for teachers to use in the preschool classroom with children to help to integrate critical AI and digital literacy skills into STEM learning (focusing on topics such as life cycles, habitats, and forces and motion). Further, given the fast pace at which new technology is being developed, it seems that teachers may benefit from professional development workshops aimed at understanding how they can integrate AI into STEM activities as an opportunity to teach such critical thinking skills for young children even before the onset of formal schooling.

### 4.2. Limitations and Future Directions

There are several limitations and future directions of this work. First, across both studies, we recruited families by posting advertisements on our lab Facebook page and sending flyers to local libraries, children’s museums, and play centers around the Northeast area. Further data for the studies were collected on Zoom. Thus, participants often needed to have access to a phone, tablet or computer to participate in the study. As a result, this may have limited the sample for both studies.

Second, a limitation of this work is that children’s experience with and familiarity with digital voice assistants and their past experiences at school may influence their preference for and trust of explanations provided by teachers and peers as sources of scientific information. It is plausible children may have been thinking about *Siri* on phones (or other digital voice assistants) at home. Importantly, all children included in the studies had some familiarity with digital voice assistants. Indeed, preliminary analyses across both studies revealed that children were most familiar with *Amazon’s* Alexa, followed by *Apple’s* Siri and *Google Home*, with no differences by condition.

Third, data from both studies focused on 4-, 5- and 6-year-olds; however, given research findings examining age-related differences in children’s trust in digital technologies including digital voice assistants and the internet (Girouard & Danovitch, 2026; Tong et al., 2025; Wang et al., 2019), future research should focus on 7- and 8-year-olds to further examine how they consider explanation quality when evaluating scientific information from digital sources.

Fourth, at the beginning of the experiment, the experimenter provided the child with a brief description of digital voice assistants, showed pictures of the branded devices, and the DVA was selected based on the device the children “know the most about,” ensuring baseline familiarity. In contrast, the teacher, however, is an abstract “teacher who teaches in your school,” and the peer is “a child like you,” both represented by pictures rather than real, known individuals. As noted above, this familiarity manipulation was not symmetric across informants, which may have impacted children’s preferences. Further, it is also possible that the children might assume that DVAs are the main focus of the study and feel compelled to select them. One possible direction for future work is to conduct research on children’s active use of digital voice assistants in everyday environments such as home or “science areas” in classroom settings. Unlike experimental work that often consists of direct prompts, such naturalistic observations would allow us to better understand how children spontaneously decide to engage with technological informants for scientific information.

## 5. Conclusions

In summary, during early childhood, digital media is shaping how children acquire scientific knowledge in the world around them. Our research provides evidence that 4-, 5- and 6-year-olds prefer to seek scientific information and trust explanations from digital voice assistants over peers (Experiment 1) or classroom teachers (Experiment 2). By understanding how children learn with and through digital technologies in the domain of science, we can design future interventions that leverage conversational AI to further enhance children’s science engagement and critical thinking skills during the early childhood years.

## Figures and Tables

**Table 1:** Children’s race and ethnicity (as reported by caregivers) for Experiment 1.

Race and Ethnicity	Number (Percent of Total Sample)
American Indian/Alaska Native	1 (0.7%)
Asian	14 (9.79%)
Asian, Hispanic/Latino	1 (0.7%)
Asian, White	9 (6.29%)
Black	12 (8.39%)
Black, Hispanic/Latino	1 (0.7%)
Black, Hispanic/Latino, White	1 (0.7%)
Black, White	4 (2.8%)
Black, American Indian or Alaska Native, White	1 (0.7%)
Hispanic/Latino	6 (4.2%)
Hispanic/Latino, White	7 (4.9%)
Hispanic/Latino, American Indian or Alaska Native, White	1 (0.7%)
South Asian	1 (0.7%)
White	80 (55.94%)
White, Middle Eastern	2 (1.4%)
Not Reported	2 (1.4%)

**Table 2:** Caregiver demographic information for Experiment 1.

Demographic Characteristics		Number (Percent of Sample)
Education Level	Less than High School	2 (1.4%)
	High School or GED	6 (4.2%)
	Some College	17 (11.89%)
	Associate’s	7 (4.9%)
	Bachelor’s	45 (31.47%)
	Master’s	51 (35.66%)
	Professional Degree or Doctorate	14 (9.79%)
	Not Reported	1 (0.7%)
Income	Under $25,000	10 (6.99%)
	$25,000–$50,000	17 (11.89%)
	$50,000–$74,999	20 (13.99%)
	$75,000–$99,000	22 (15.38%)
	$100,000–$149,000	21 (14.69%)
	$150,000–$199,000	23 (16.08%)
	$200,000–$249,000	10 (6.99%)
	$250,000–$299,000	4 (2.79%)
	Over $300,000	6 (4.2%)
	Not Reported	10 (6.99%)

**Table 3:** Science questions and explanations used in Studies 1 (DVA vs. peer) and 2 (DVA vs. teacher) ask and endorse phase (adapted from Oranç & Küntay, 2020; Corriveau & Kurkul, 2014).

Science Topic	Ask Questions (Part 1)	Endorse Question (Part 2)
**Airplanes**	If you are going to find out why airplanes can fly, which would you like to ask, [Alexa/Siri/Google] or your teacher? Why?	**Circular Explanation**: Airplanes can fly because they go at a speed fast enough to keep them in the air. **Noncircular Explanation**: Airplanes can fly because they are powered by engines, and they have wings that give them lift. **Endorse Question**: Do you like what [Alexa/Siri/Google] or your teacher [the child] said? Why?
**Electricity**	If you are going to find out why lights turn on when you flip the switch, which would you ask, Alexa/Siri/Google or your teacher? Why?	**Circular Explanation**: The lights turn on because the switch is turned on, and it gives us light. **Noncircular Explanation**: Lights turn on because the circuit is complete, and electricity flows to turn on the light. **Endorse Question**: Do you like what [Alexa/Siri/Google] or your teacher [the child] said? Why?
**Fish**	If you are going to find out how fish breathe under water, which would you ask, [Alexa/Siri/Google] or your teacher? Why?	**Circular Explanation**: Fish breathe under the water and fish do not live or move around on land. **Noncircular Explanation**: Fish take in water through their gills and dissolve oxygen from water into their blood. **Endorse Question**: Do you like what [Alexa/Siri/Google] or your teacher [the child] said? Why?
**Rain**	If you are going to find out why it rains, which would you ask, Alexa/Siri/Google or your teacher? Why?	**Circular Explanation**: It rains because water falls from the sky and gets us wet. **Noncircular Explanation**: It rains because the clouds fill with water and get too heavy. **Endorse Question**: Do you like what [Alexa/Siri/Google] or your teacher [the child] said? Why?
**Flowers/Trees**	If you were going to find out why trees and flowers grow, which would you ask, Alexa/Siri/Google or your teacher? Why?	**Circular Explanation**: Trees and flowers grow because their stems get longer and longer, and they get taller. **Noncircular Explanation**: Trees and flowers grow because we feed them water, and the sun gives them light **Endorse Question**: Do you like what [Alexa/Siri/Google] or your teacher [the child] said? Why?

**Table 4:** Sample justification responses for science ask and endorse trials.

Code	Definition	Examples
**Attribute**	Responses that discuss an attribute about the informant (e.g., kindness, warmth, love, or helping behavior).	“Because my teacher loves me so much.” “Because I love Google [home].”
**Knowledge**	Responses that refer to the informant knowing specific content or the answer to the question.	“Because she knows how trees grow.” “Because Alexa knows a lot.”
**Prior Knowledge**	Responses that refer to what the child has learned before or their own knowledge about the topic.	“Because that’s what I know.” “Because I just said that before.”
**Making Sense/Understand**	Responses refer to the child indicating that the explanation “made sense” or they that they understood it.	“Because I understand it.” “That made more sense.”
**Truth/Right**	Responses that indicate one informant is telling the truth or is right.	“Alexa’s right.” “It is actually true.”
**Repetition**	Responses repeated key words from the science question or the informant’s explanation.	“Fish” “Airplane!”
**Better**	Responses indicated that one informant is better at answering questions or provided a better explanation.	“Because she is better at it.” “Because it has a better answer.”
**Status as Informant**	Responses referred to characteristics of digital voice assistants or human characteristics of the teacher.	“Because it is a phone device.” “Because she’s a human and Alexa’s not a human.”
**I don’t know**	Responses that included “I don’t know.”	“I don’t know.”
**No Answer/Nonsense**	The child did not respond or gave an irrelevant response.	

**Table 5:** Sample justification responses for explicit judgement questions.

Code	Definition	Examples
**Attribute**	Responses that discuss an attribute about the informant (e.g., kindness, warmth, love, or helping behavior).	“Because Google [home is your friend].”
**Knowledge**	Responses that refer to the informant knowing specific content or the answer to the question.	“She’s more smarter than five billion teachers.”
**Prior Experience**	Responses that refer to how many times the child chose an informant during the science ask and endorse trials.	
**Explanation Quality**	Responses that refer to the explanations provided by the informant.	
**Accuracy/Inaccuracy**	Responses that indicate how accurate informants were in responding to questions.	“Because she answered all of the questions right.” “Siri was wrong.”
**Repetition**	Responses repeated key words from the science question or the informant’s explanation.	“She said airplanes have speed so it can stay in the air.”
**Better**	Responses indicated that one informant is better at answering questions or provided a better explanation.	“Because I like what the teacher said better.”
**Status as Informant**	Responses referred to characteristics of digital voice assistants or human characteristics of the teacher.	“Because Teachers mainly do science and Alexa doesn’t.” “Because she’s a human and Alexa’s not a human.”
**I don’t know**	Responses that included “I don’t know.”	“I don’t know.”
**No Answer/Nonsense**	The child did not respond or gave an irrelevant response.	

**Table 6:** Logistic regression models examining whether age predicts children’s choice of DVA vs. a peer (holding condition constant).

Model	Age (4-Year-Olds as Reference Group)	Odds Ratio	95% Confidence Interval
Initial Ask Preference	5-year-olds	1.37	(0.57, 3.33)
	6-year-olds	0.85	(0.35, 2.09)
Initial Endorsement	5-year-olds	1.15	(0.50, 2.64)
	6-year-olds	0.77	(0.32, 1.85)
Total Science Ask	5-year-olds	2.37	(0.97, 6.05)
	6-year-olds	1.42	(0.57, 3.63)
Total Science Endorse	5-year-olds	2.38	(1.00, 5.88)
	6-year-olds	0.85	(0.35, 2.09)

**Table 7:** Distribution of justifications for total science ask, total science endorsement, and explicit judgement questions in Experiment 1 (DVA vs. peer).

Justification Code	Total Science Ask	Total Science Endorsement	Explicit Judgement: Which Is Better?
Attribute	7 (0.049)	7 (0.049)	8 (0.056)
I don’t know	52 (0.364)	56 (0.392)	51 (0.357)
Knowledge	41 (0.287)	13 (0.091)	32 (0.224)
Matches prior knowledge	2 (0.014)	15 (0.105)	NA
No pattern	17 (0.119)	22 (0.154)	NA
Nonsense	15 (0.105)	13 (0.091)	9 (0.063)
Status as informant	6 (0.042)	1 (0.007)	9 (0.054)
Factually correct	NA	11 (0.077)	NA
Is better	NA	1 (0.006)	10 (0.070)
Making sense/understanding	NA	1 (0.007)	NA
Accuracy/inaccuracy	NA	NA	13 (0.091)
Explanation quality	NA	NA	7 (0.049)
Picked most or least of the time	NA	NA	5 (0.035)
Repeat science information	NA	NA	2 (0.014)
Repetition question word	2 (0.014)	1 (0.007)	NA
No answer	NA	1 (0.007)	NA

*Note*: Numbers reported as count (proportion).

**Table 8:** Children’s race and ethnicity (as reported by caregivers) for Experiment 2.

Race and Ethnicity	Number (Percent of Total Sample)
American Indian/Alaska Native, White	2 (1.2%)
Asian	19 (11.38%)
Asian, White	8 (4.79%)
Asian, Native Hawaiian or Other Pacific Islander, White	2 (1.19%)
Asian, Native Hawaiian or Other Pacific Islander, White, Puerto Rican	1 (0.59%)
Asian, Middle Eastern	1 (0.59%)
Black	10 (5.99%)
Black, Hispanic/Latino	4 (2.4%)
Black, Hispanic/Latino, White	1 (0.59%)
Black, White	9 (5.39%)
Hispanic/Latino	8 (4.79%)
Hispanic/Latino, White	7 (4.19%)
Jewish	1 (0.59%)
White	90 (53.89%)
White, Middle Eastern	2 (1.19%)
White, Hispanic/Latino, Native Hawaiian	1 (0.59%)
Not Reported	1 (0.59%)

**Table 9:** Caregiver demographic information (highest education level achieved and income) for Experiment 2.

Demographic Characteristics		Number (Percent of Sample)
Education Level	Less than High School	0 (0%)
	High School or GED	8 94.79%)
	Some College	17 (10.18%)
	Associate’s	11 (6.59%)
	Bachelor’s	53 (31.74%)
	Master’s	51 (30.54%)
	Professional Degree or Doctorate	23 (14.37%)
	Not Reported	3 (1.8%)
Income	Under $25,000	11 (6.59%)
	$25,000–$50,000	21 (12.57%)
	$50,000–$74,999	18 (10.77%)
	$75,000–$99,000	29 (17.37%)
	$100,000–$149,000	31 (18.56%)
	$150,000–$199,000	18 (10.77%)
	$200,000–$249,000	13 (7.78%)
	$250,000–$299,000	8 (4.79%)
	Over $300,000	5 (2.99%)
	Not Reported	13 (7.78%)

**Table 10:** Logistic regression models examining whether age predicts children’s choice of DVA vs. a teacher (holding condition constant).

Model	Age (With 4-Year-Olds as Reference Group)	Odds Ratio	95% Confidence Interval
Initial Ask Preference	5-year-olds	0.50	(0.27, 0.98)
	6-year-olds	0.82	(0.38, 1.78)
Initial Endorsement	5-year-olds	1.75	(0.75, 4.21)
	6-year-olds	2.21	(0.96, 5.29)
Total Science Ask	5-year-olds	0.53	(0.23, 1.21)
	6-year-olds	0.76 *	(0.34, 1.70)
Total Science Endorse	5-year-olds	0.68	(0.26, 1.74)
	6-year-olds	0.85 *	(0.35, 2.09)

* *p* < 0.05.

**Table 11:** Distribution of justifications for total science ask, total science endorsement, and explicit judgement questions in Experiment 2 (DVA vs. teacher).

Justification Code	Total Science Ask	Total Science Endorsement	Explicit Judgement: Which Is Better?
Attribute	6 (0.036)	13 (0.078)	6 (0.036)
I don’t know	41 (0.248)	52 (0.311)	44 (0.263)
Knowledge	58 (0.352)	17 (0.102)	31 (0.186)
Matches prior knowledge	1 (0.006)	13 (0.078)	NA
No pattern	16 (0.097)	26 (0.156)	NA
Nonsense	39 (0.236)	29 (0.174)	31 (0.186)
Status as informant	2 (0.012)	NA	9 (0.054)
Factually correct	2 (0.012)	12 (0.072)	NA
Is better	NA	1 (0.006)	16 (0.096)
Making sense/understanding	NA	4 (0.024)	NA
Accuracy/inaccuracy	NA	NA	13 (0.078)
Explanation quality	NA	NA	14 (0.263)
Picked most or least of the time	NA	NA	1 (0.006)
Repeat science information	NA	NA	2 (0.012)

*Note*: Numbers reported as count (proportion).

## Data Availability

Data are available from the first author upon request.

## Supplementary Materials

The following supporting information can be downloaded at: https://www.mdpi.com/article/10.3390/bs16050661/s1.
