# Supplementary material for: How Do Children Evaluate Scientific Explanations Provided by Digital Voice Assistants, Teachers, and Peers?
Source: Behav Sci (Basel). 2026 Apr 27;16(5):661. doi: 10.3390/bs16050661 (PMC13203908; doi:10.3390/bs16050661)
Supplement: Supplementary file 1 [file behavsci-16-00661-s001.zip › behavsci-4160704-supplementary.pdf]

Analytic Plan for Study 1 and Study 2

Our analytic plan was the same for Study 1 and Study 2, with the only difference being that Study 1 compared Peer to DVA and Study 2 compared Teacher to DVA. In Condition 1, the DVA provides a noncircular explanation (and therefore, the Teacher/Peer provides a circular explanation); whereas, in Condition 2, the DVA provides a circular explanation (and therefore, the Teacher/Peer provides a noncircular explanation). Thus, for each condition, the informant and the explanation quality were intertwined, making it difficult to untangle the two factors. In order to untangle the factors, we conducted binomial exact tests for each of our outcome variables of interest (Initial Science Ask Preference, Initial Science Endorsement, Total Science Ask Preference, Total Science Endorsement, and Explicit Judgment). We first conducted binomial exact tests to determine whether the proportion of children with a DVA preference compared to a teacher or peer preference differed from chance (0.5). We then split the data into children who chose the DVA and children who chose the teacher or peer in order to examine differences in decision making by explanation type (circular or noncircular).

Finally, we were interested in whether there were age-related differences controlling for Condition, so we conducted logistic regressions on each of our DVs of interest, with Age as the predictor of interest and Condition as a covariate.
